# Construction of a Xylanase A Variant Capable of Polymerization

**DOI:** 10.1371/journal.pone.0025388

**Published:** 2011-09-23

**Authors:** Veronika Szabó, Adél Muskotál, Balázs Tóth, Marko D. Mihovilovic, Ferenc Vonderviszt

**Affiliations:** 1 Bio-Nanosystems Laboratory, Faculty of Information Technology, Research Institute of Chemical and Process Engineering, University of Pannonia, Veszprém, Hungary; 2 Agricultural Research Institute, Hungarian Academy of Sciences, Martonvásár, Hungary; 3 Institute of Applied Synthetic Chemistry, Vienna University of Technology, Vienna, Austria; 4 Institute of Enzymology, Hungarian Academy of Sciences, Budapest, Hungary; University of Crete, Greece

## Abstract

The aim of our work is to furnish enzymes with polymerization ability by creating fusion constructs with the polymerizable protein, flagellin, the main component of bacterial flagellar filaments. The D3 domain of flagellin, exposed on the surface of flagellar filaments, is formed by the hypervariable central portion of the polypeptide chain. D3 is not essential for filament formation. The concept in this project is to replace the D3 domain with suitable monomeric enzymes without adversely affecting polymerization ability, and to assemble these chimeric flagellins into tubular nanostructures. To test the feasibility of this approach, xylanase A (XynA) from *B. subtilis* was chosen as a model enzyme for insertion into the central part of flagellin. With the help of genetic engineering, a fusion construct was created in which the D3 domain was replaced by XynA. The flagellin-XynA chimera exhibited catalytic activity as well as polymerization ability. These results demonstrate that polymerization ability can be introduced into various proteins, and building blocks for rationally designed assembly of filamentous nanostructures can be created.

## Introduction

Here we address the problem how to combine self-assembling ability of structural proteins with the catalytic capabilities of enzymes. Long helical filaments of bacterial flagella are made of thousands of flagellin (FliC) subunits [1]. Flagellin from *S. typhimurium* is composed of 494 amino acid residues. Both terminal regions of the molecule, residues 1–66 and 450–494, are disordered in the monomeric state, and become folded upon filament formation [2]. Flagellin subunits are synthesized in the cell and exported by the flagellum-specific export apparatus from the cytoplasm to the site of assembly at the distal end of the growing filament [3]. They are assumed to move through the narrow - 20–25 Å wide - central channel of the flagellum as partially unfolded monomers, and acquire their folded conformation in the cavity below the HAP2 cap attached to the tip of filaments [4]. X-ray diffraction and cryo-EM studies revealed that the highly conserved terminal regions of flagellin are involved in subunit interactions in the filament core, while the hypervariable central portion of the polypeptide chain forms the D3 domain exposed on the filament surface [5], [6]. Our studies demonstrated that D3 is a structurally independent part of flagellin which has no significant role in the construction of the filament structure [7]. This observation suggests that the D3 domain can be replaced by foreign proteins without influencing polymerization ability.

Xylanases (β-1,4-xylan xylanohydrolases; EC 3.2.1.8) perform hydrolysis of the β-1,4-glycosidic bonds of xylan, the major constituent of hemicellulose in the plant cell wall, and are important enzymes in the paper, food and animal feed industries [8]. The high resolution crystal structure of the active form of xylanase A (XynA) from *Bacillus subtilis* contains an α-helix and two twisted β-sheets forming a jellyroll fold [9]. Its active form is composed of 185 amino acids.

The concept of our work is to engineer flagellin to give it catalytic functionalities by replacing the D3 domain with suitable monomeric enzymes without adversely affecting polymerization ability, and to assemble these chimeric flagellins into tubular nanostructures. In this exploratory study, the active form of XynA from *B. subtilis* was introduced into the central part of flagellin. It was investigated whether the fusion protein preserved the essential functional properties of both of its parental proteins.

## Materials and Methods

### Construction of the flagellin-xylanase A fusion gene

A deletion mutant of flagellin lacking the D3-domain-coding region (ΔD3-FliC) was created as described previously [7] using the pKOT1 construct (kind gift from Kenji Oosawa; Nagoya University, Japan). Furthermore, the *Pme*I cleavage site was replaced by a cassette containing the recognition sites of *Xho*I, *Age*I, *Xma*I and *Sac*I enzymes using site directed mutagenesis following the manufacturer's instruction (QuikChange II Site-Directed Mutagenesis Kit, Stratagene). This modified ΔD3-FliC gene was amplified using forward primer 5′-ACATCATATGATGGCACAAGTCATTAATACAAACAGCC-3′ and reverse primer 5′-ACATGGATCCTTAACGCAGTAAAGAGAGGACGTTTTG-3′, and then it was inserted into a pET19b vector (Novagene) using the *Nde*I and *Bam*HI sites. To minimize the number of extra N-terminal amino acids which may disturb polymerization ability of the expressed protein, the original N-terminal 10 His-tag was shortened to 6-His and the enterokinase cleavage site together with the *Nde*I site was removed from the construct applying 2-step site directed mutagenesis. This redesigned plasmid served as a cloning vector for the production of the N-terminal His_6_-tagged version of FliC-xylanase A. The gene of xylanase A was synthesized by Genscript Corporation (Piscataway, NJ, USA) based on the protein sequence of xylanase A from *Bacillus subtilis* (UniProt P18429), and it was inserted into the ΔD3-FliC gene between the *Xho*I and *Sac*I restriction sites using the Rapid DNA Ligation Kit (Fermentas). The obtained plasmid construct (containing the gene of FliC(XynA)) was transformed into *E. coli* TOP10 cells (Invitrogen) using the CaCl_2_ procedure, checked by *Xho*I and *Sac*I digestion, and sequenced to confirm the presence of the desired DNA sequence and the correct reading frame. The FliC(XynA) construct contained a His_6_-tag and a few flanking amino acids (the MGHHHHHHGGS segment) attached to its N-terminus.

### Protein expression and purification

The pET19b-based construct was transformed into BL21-CodonPlus (DE3)-RIL cells to overexpress the His-tagged FliC(XynA) fusion protein. 100 ml overnight culture was inoculated into 2 L of LB broth, supplemented with ampicillin (100 µg/ml) and incubated at 37°C to reach an OD_600_ of 0.6. After addition of IPTG to a final concentration of 0.5 mM, incubation was continued at 20°C for another 12 hours. The bacterial pellet was collected by centrifugation at 4000 g for 30 min and stored at −20°C.

Purification of FliC(XynA) was performed as follows: the bacterial pellet from 2 L culture was resuspended in 80 ml 20 mM sodium phosphate buffer containing 500 mM NaCl and 50 mM imidazole, pH = 7.4, and 2 mini Complete tablets (EDTA-free) were added to inhibit unwanted proteolytic degradation. The sample was sonicated on ice to lyse the cells for 1 min 4 times at power of 10W. The solution was centrifuged at 50000 rpm 30 min at 4°C to remove cell debris and aggregates. The supernatant was prepurified on a SP FF cation exchange column (Amersham) to remove contaminating proteases [10]. Then the flow-through fraction was applied to a 5 ml HisTrap Chelating Ni-affinity column (Amersham Pharmacia Biotech). The absorbed proteins were eluted using a linear imidazole gradient (50 mM–200 mM), the His_6_-tagged FliC(XynA) protein was eluted at about 150 mM imidazole. After dialysis against 20 mM Tris-HCl buffer (pH 7.8) at 4°C, samples were further purified by anion-exchange chromatography on a MonoQ HR10/10 column. The elution buffer was 20 mM Tris-HCl (pH 7.8) and 0 to 0.2 M NaCl/20 min gradient was used at a 2 ml/min flow rate. Pure fractions were collected and dialyzed at 4°C against 20 mM Tris-HCl (pH 7.8) buffer, containing 150 mM NaCl, and stored at −20°C.

Flagellin was purified as described previously [2].

Protein concentrations were determined from absorption measurements at 280 nm using molar extinction coefficients (ε_280_ = 17880 M^−1^ cm^−1^ for flagellin, ε_280_ = 82850 M^−1^ cm^−1^ for XynA, and ε_280_ = 93280 M^−1^ cm^−1^ for FliC(XynA)) calculated from the known aromatic amino acid contents of the molecules [11]. Purity of protein samples was checked using SDS-PAGE followed by Coomassie blue R-250 staining.

### Expression and purification of XynA

N-terminal His-tagged xylanase A was also cloned, overexpressed and purified in order to determine reference enzyme activity. For this construct, the gene of xylanase A from *B. subtilis* was inserted into a pET19b vector using *Xho*I and *Bam*HI restriction enzymes. Recombinant XynA was overexpressed in *E. coli* BL21-CodonPlus (DE3)-RIL cells and purified by Ni-affinity chromatography on a HisTrap column. XynA eluted at about 150 mM imidazole concentration. Collected fractions were dialyzed against PBS buffer and stored at −20°C.

### Limited proteolysis

Limited proteolysis by trypsin was performed in 10 mM phosphate buffer, pH 7.0, containing 150 mM NaCl at room temperature at a protein concentration of 0.8 mg/ml. In a typical digestion experiment, the protease was added to the protein solution at a 1∶300 or 1∶30 (w/w) ratio. At various times 10 µl samples were taken and mixed with 10 µl electrophoretic sample buffer, followed immediately by heating in a boiling water bath for three minutes. Electrophoresis was performed on 12.5% or 15% (w/v) polyacrylamide slab gels.

### Assay of xylanase activity

Xylanase activity was determined by measuring the increase in reducing groups during the enzymatic hydrolysis of xylan by the dinitrosalicylic acid method of Bernfeld [12]. The reaction mixture consisting of 250 µl of 1% xylan solution in 50 mM phosphate buffer, pH 6.0, and 100 µl of enzyme solution at 4.2 µM concentration was incubated at 37°C for 10 min. After addition of 375 µl DNSA solution, the reaction mixture was boiled at 100°C for 20 minutes. The amount of reducing sugars was determined by absorbance measurement at 575 nm. The standard xylose curve was calibrated with D-xylose within the range of 0–200 µg/ml. Measurements were repeated three times and averaged.

### Polymerization experiments

The polymerizing ability of the FliC(XynA) fusion protein was investigated by inducing nucleation and polymerization by ammonium sulfate (AS) [13]. Protein solutions of 1 to 1.5 mg/ml were prepared in 20 mM Tris-HCl (pH 7.8) containing 150 mM NaCl and 4 M AS was added to various final concentrations in the range of 0.4 M to 0.8 M. Filament formation was observed after 24 h of incubation at room temperature. For copolymerization of flagellin and FliC(XynA), monomer solutions of Salmonella SJW1660 flagellin capable of forming L-type straight filaments [1] and FliC(XynA) in 20 mM Tris-HCl, 150 mM NaCl (pH 7.8) were mixed at various protein ratios in the range of 1∶1 to 1∶5 (w/w), and polymerized by the addition of AS to various final concentrations. The mixtures were left at room temperature for 2 days to accomplish polymerization. Filaments were observed by dark-field optical microscopy with an Olympus BX50 microscope. They were also visualized by atomic force microscopy using a DigiScope1000 (Aist-NT) microscope, the scanned area was typically 4 µm ×4 µm.

## Results

### Design and preparation of the FliC(XynA) fusion construct

**Figure 1:**
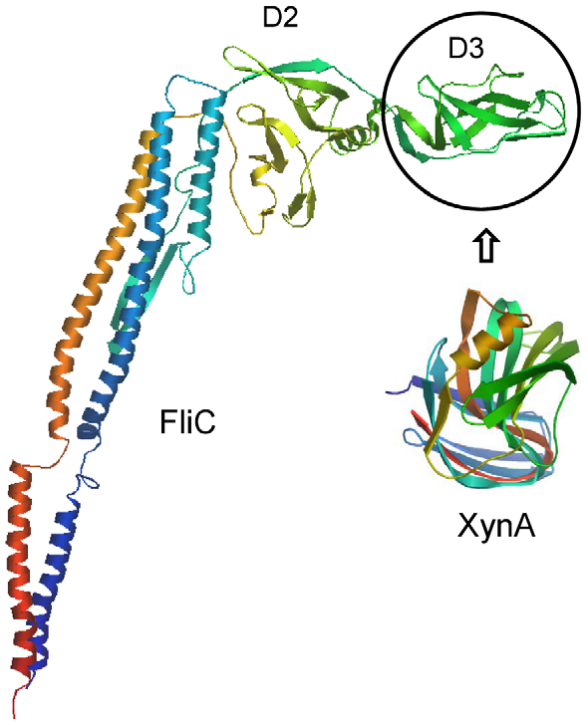
Design of the FliC(XynA) fusion construct. The hypervariable D3 domain of Salmonella flagellin (residues 190–284) was replaced by xylanase A from *B. subtilis*. The C_α_ backbone trace of flagellin and XynA is rainbow-colored from blue to red representing the sequence from the NH_2_- to COOH terminus.

This work aims at the creation of a flagellin-based fusion protein in which the D3 domain of FliC is replaced by the xylanase A enzyme from *B. subtilis* (Fig. 1). In order to obtain a fusion protein which preserves both the catalytic activity of XynA and the polymerization ability of FliC, it is essential to use appropriate linkers for insertion of XynA into the middle portion of flagellin. The linkers should allow proper folding of both partners. According to the high resolution structural model of Salmonella flagellin [5], [6], the resulting inner ends of the molecule upon removal of D3 are close to each other, separated by only 6 Å, and are formed by two extended anti-parallel chains. The N- and C-terminal residues of XynA are also closely separated by only 5.4 Å [9], however, the terminal chains are almost perpendicular to each other. Upon inspection of the 3D structures (PDB codes: 1UCU, 1XXN; [6], [9]) we concluded that a short, 2-residue long linker at both ends of XynA may suffice.

In a previous work, a D3 deleted variant of Salmonella flagellin (ΔD3_FliC) was created in which the ends of the discontinuous D2 domain were connected by the short GLNSA segment [7]. Now, this segment was replaced by a longer one (LETGPGEL) which was designed to be encoded by a gene cassette containing the recognition sites of *Xho*I, *Age*I, *Xma*I and *Sac*I restriction enzymes to facilitate insertion of foreign genes. We used the *Xho*I and *Sac*I restriction sites for insertion of the XynA gene which resulted in the LE and EL linker segments at the N- and C-terminus of the inserted XynA, respectively. The fusion gene of FliC(XynA) was ligated into a modified pET19b-based plasmid which allows overexpression of an N-terminal His_6_-tagged version of the cloned recombinant protein.

The FliC(XynA) fusion construct was produced in IPTG-inducible *E.coli* BL21-CodonPlus (DE3)-RIL cells. It appeared in the cytosolic fraction in significant amounts as judged by SDS-PAGE. Cells were disrupted by sonication and the fusion protein was purified from the soluble fraction by combining Ni-affinity and ion-exchange chromatography. Typically, 10–15 mg purified FliC(XynA) was obtained from 1L culture. The sample was dialyzed against 20 mM Tris-HCl (pH 7.8) buffer, containing 150 mM NaCl, and stored at −20°C.

### Investigation of polymerization properties

**Figure 2:**
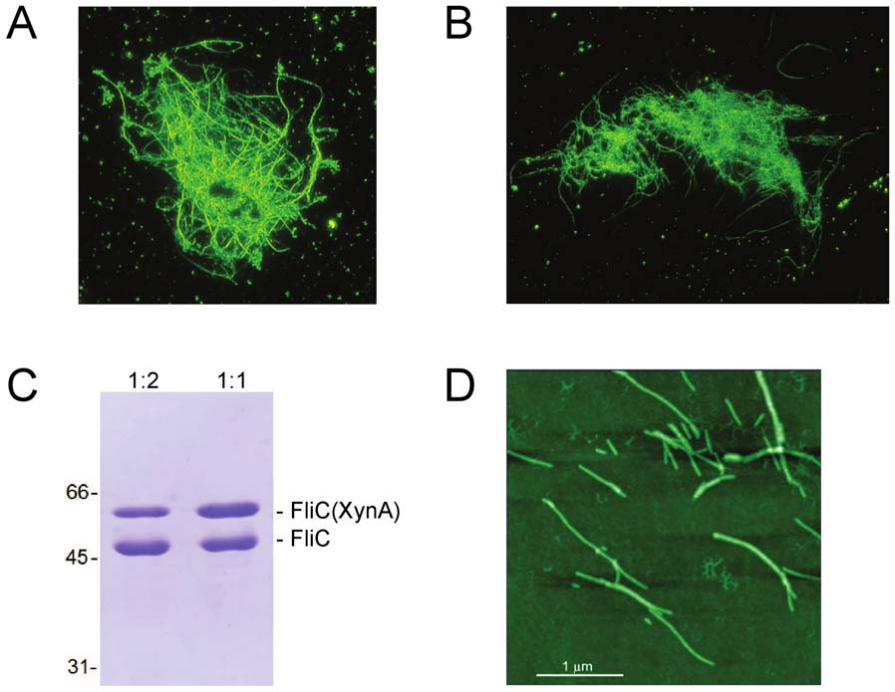
Products of the polymerization of FliC(XynA) subunits. Dark-field light micrographs of (A) FliC(XynA) filaments (0.6 M AS) and (B) copolymers of flagellin and FliC(XynA) at 1∶1 (w∶w) mixing ratio (0.4 M AS). Polymerization experiments were done in 20 mM Tris-HCl (pH 7.8) containing 150 mM NaCl at 1 to 1.5 mg/ml protein concentration, and 4 M AS was added to a final concentration indicated in parentheses to initiate filament formation. (C) SDS/PAGE analysis of copolymer samples polymerized at FliC(XynA) to flagellin ratios of 1∶1 and 1∶2. After AS-induced (0.6 M) polymerization, samples were centrifuged and the pellet was dissolved in 20 mM Tris-HCl (pH 7.8) buffer containing 150 mM NaCl. The molecular masses of flagellin and FliC(XynA) are 51.5 kDa and 64 kDa, respectively. Band intensities determined by densitometry were {49.3 (1∶1); 50.7 (1∶2)} and {49.6 (1∶1) and 34.2 (1∶2)} for FliC and FliC(XynA), respectively. (D) Copolymers observed by atomic force microscopy.

Polymerization ability of the purified FliC(XynA) protein was investigated. Filament formation was induced by adding ammonium sulfate (AS) to a monomer solution of 1 mg/ml at a final concentration in the range of 0.4–0.8 M. We found that FliC(XynA) effectively polymerized into filaments. At 0.4 M AS, aggregates of long filaments were observed under the dark field microscope (Fig. 2A). At higher AS concentration, shorter filaments were observed (not shown). However, the stability of FliC(XynA) filaments required high precipitant concentration (>0.3 M AS), and they dissociated within a few hours after changing the buffer to 20 mM Tris-HCl (pH 7.8) containing 150 mM NaCl.

Highly stable filaments were obtained by mixed copolymerization of FliC(XynA) with flagellin at 1∶1 or 1∶2 ratios (Fig. 2B) which remained intact even after the removal of AS from the solution by spinning down the sample and dissolving the pellet in 20 mM Tris/150 mM NaCl (pH 7.8). Densitometric analysis of SDS-PAGE gels of copolymer samples revealed that FliC(XynA) was incorporated into the filaments in a proportion similar to the applied mixing ratio (Fig. 2C). Filaments were also observed by AFM (Fig. 2D). Their length was typically in the range of 200 nm –1200 nm, and they showed a straight polymorphic form.

**Figure 3:**
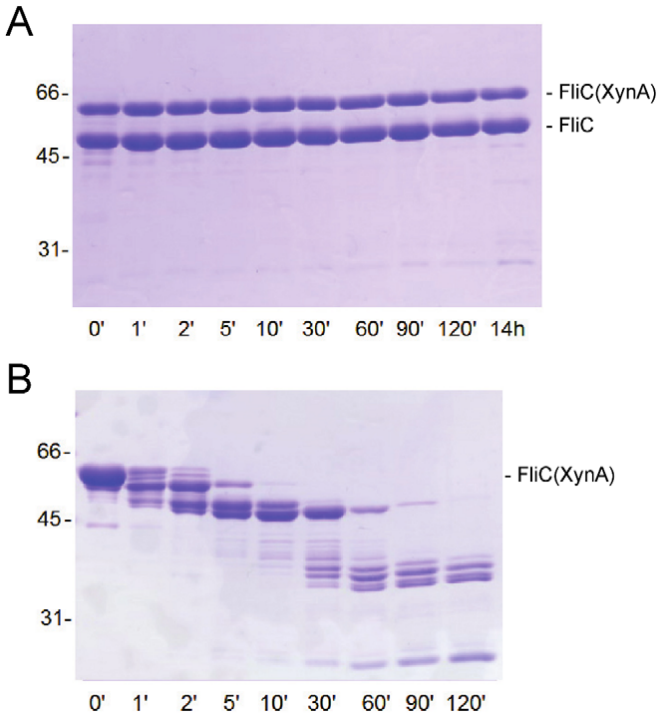
Proteolytic sensitivity of FliC(XynA) in the polymeric and monomeric form. (A) Copolymers of flagellin and FliC(XynA) obtained by 0.6 M AS at 1∶1 mixing ratio (w/w) were incubated with trypsin at a 30∶1 (w/w) ratio. (B) Proteolysis of monomeric FliC(XynA) at a protein to protease ratio of 300∶1 (w/w). At the indicated time points, portions were removed from the reaction mixtures, mixed with electrophoresis sample buffer and boiled for 3 min. Experiments were done in 20 mM Tris–HCl, 150 mM NaCl (pH 7.8) at room temperature at a 1 mg/ml protein concentration.

Structural stability of mixed copolymers was probed by proteolytic digestion. Native filaments are known to be highly resistant against tryptic hydrolysis even at room temperature [7]. On the other hand, depolymerized flagellin subunits are quickly degraded under the same conditions since their terminal regions are disordered in the monomeric state [2]. As shown in Figure 3A, FliC(XynA) containing filaments were highly resistant against tryptic degradation even at a 30∶1 (w/w) ratio. They remained essentially intact even after an overnight incubation with the protease. These results demonstrate that FliC(XynA) has the ability to form stable filaments when copolymerized together with wild type flagellin, and the XynA portion of subunits has a well-folded, compact conformation. This latter conclusion is also supported by limited proteolysis of monomeric FliC(XynA) (Fig. 3B) which reveals that the natively disordered terminal regions of the fusion protein are highly sensitive to tryptic cleavage but the central portion shows significant stability against proteolytic digestion.

### Catalytic properties of FliC(XynA)

Catalytic properties of the FliC(XynA) fusion protein in the monomeric as well as the polymeric state were studied and compared to recombinant wild type xylanase A. XynA can degrade xylan by cleavage of β-1,4-xylosidic linkages. Reducing sugars liberated during the course of hydrolysis were determined by the method of Bernfeld [12] based on absorbance measurements at 575 nm. Color development was compared to a standard xylose curve that was calibrated with D-xylose within the range of 0–200 µg/ml. Activities for the FliC(XynA) fusion protein in the monomeric and polymeric state were determined at 37°C in 50 mM phosphate buffer, pH 6.0 (Table 1). Activity measurements were done at substrate concentration significantly below the saturation level. There are two reasons for this. One is the low solubility of xylan. Another important factor is that the critical flagellin concentration for filament assembly is about 0.2 µM. To study catalytic activity of FliC(XynA) in the filamentous form we had to choose a protein concentration which ensures that the subunits are dominantly in the filament form. Color development by visual inspection clearly indicated that FliC(XynA) was enzymatically active in both the monomeric and polymeric forms. As shown in Table 1, monomeric FliC(XynA) exhibited a xylan degrading activity similar to that of the recombinant wild-type enzyme. Mixed copolymers were used to investigate the enzymatic activity of FliC(XynA) subunits in the filamentous state. The amount of FliC(XynA) subunits incorporated into the copolymer was estimated by densitometric analysis of SDS-PAGE gels of the corresponding filaments. Our data indicate that the activity of polymeric FliC(XynA) was virtually identical to that of the monomeric form. These experiments demonstrate that XynA maintains its catalytic activity upon insertion into the variable central part of flagellin, and filament formation has no significant influence on the catalytic efficiency of subunits.

**Table 1:** Catalytic activity of recombinant xylanase A and the FliC(XynA) fusion protein in the monomeric and polymeric forms.

Sample	OD_575_	D-xylose deliberated (µg/ml)
native xylanase A	0.175	26.7
FliC(XynA) monomer	0.156	23.8
FliC/FliC(XynA) copolymer 2∶1 (w/w)	0.153	23.3
FliC/FliC(XynA) copolymer 1∶1 (w/w)	0.163	24.9

Measurements were done at 1.2 µM enzyme (catalytic unit) concentration in 50 mM phosphate buffer (pH 6.0) at 37°C. The amount of FliC(XynA) subunits incorporated into the copolymers was estimated by densitometric analysis of SDS-PA gels of filament samples.

## Discussion

Nanobiocatalysis, in which enzymes are incorporated into nanostructured materials, has emerged as a rapidly growing area [13], [14]. Fabrication of nanostructured biocatalysts via self-assembly is an attractive approach which has the potential to allow fabrication of multi-enzymes nanoscale factories. In an early attempt, Baxa et al. [15] fused the prion domain of the amyloidogenic Ure2p protein with various enzymes and demonstrated that the structures of the appended enzymes were modulated very slightly by incorporation into filaments. Using a similar approach, cytochrome b562 was also successfully displayed on the surface of SH3 amyloid fibrils [16].

We address the problem how to fabricate self-assembling tubular nanostructures displaying various catalytic functionalities. In this work we created a fusion construct of a polymerizable protein, flagellin, and the xylanase A enzyme in such a way that the essential functional properties of both partners were preserved. XynA was inserted into the hypervariable central portion of flagellin replacing the D3 domain which has no significant role in filament formation [7]. The FliC(XynA) fusion protein exhibited polymerization ability and catalytic activity as well. The rate of xylan degradation by FliC(XynA) both in the monomeric and polymeric state was similar to that observed by the recombinant XynA enzyme. The FliC(XynA) fusion protein readily formed filaments (upon addition of AS) which were, however, stable only in the presence of the AS precipitant (>0.3 M), and depolymerized slowly under physiological-like conditions. Nevertheless, highly stable filaments were obtained by mixed copolymerization of FliC(XynA) with intact flagellin at various ratios (in the range of 1∶1 – 1∶5). These copolymers remained stable upon removal of the AS and were highly resistant to proteolytic digestion.

The observed polymerization properties of FliC(XynA) were reminiscent to those of flagellin variants containing small truncations in the N-terminal part of the molecule [17] which also formed straight filaments of decreased stability. In the core of the flagellar filament there is a pair of concentric double-tubular structures composed of the inner-tube and the outer-tube [6]. Our previous studies have shown that the inner-tube structure is quite independent from the outer-tube structure [18]–[19]. The main driving force behind polymerization is the interaction between the outer-tube domains of subunits, and the basic structure of the flagellar filament can be formed with the interactions in the outer-tube alone. The very terminal regions of flagellin are known to construct the inner tube and the wall of the narrow central channel of flagellar filaments [6], [18]. They are not essential for polymerization, but they do play an important role in stabilization of the filament structure [17]. Cryo-EM studies revealed that small terminal truncations of subunits resulted in misfolding of the inner tube structure [18], and destabilization of the filament. The similar polymerization properties of FliC(XynA) suggest conformational strains in the filament core. Close inspection of the available 3D structure of the flagellar filament [6] revealed that the wall of the central channel is composed of the helical C-terminal portion of subunits while their N-terminal part is buried under this layer. The FliC(XynA) fusion protein contains a His_6_-tag and a few additional flanking amino acids attached to its N-terminus. It seems that these extra N-terminal residues cannot be readily accommodated and imposes a conformational strain on the structure of the inner-tube, reflected in the decreased stability. Copolymerization with intact flagellin may significantly relieve these distortions. Work is under way to develop a FliC(XynA) variant in which the fusion tag used for easy purification is attached to the C-terminus or removable by selective proteolytic digestion.

The central part of flagellin has been a favorite target site for insertion of foreign peptides or domains [20]–[22]. The primary aim of these studies was to display the inserted unit outside the cell on the surface of flagellar filaments, and study its interaction with specific ligands or characterize its immunogenic properties. However, this kind of flagellar display has severe limitations. Subunits of flagellar filaments are exported by the flagellum-specific export machinery through the narrow central channel of filaments [3]. In the majority of cases flagellin-based fusion constructs are not exported by the flagellar export system because of size limitations or other compatibility problems (related to the amino acid composition, surface properties, the presence of disulfide bonds etc.).

Notwithstanding, these problems do not mean that fusion proteins not applicable to flagellar display are unable to form filaments in vitro. As we realized, these fusion proteins can be overexpressed in bacteria, and after purification from the cells they are usable to build filamentous assemblies. This idea was tested in this study by exploring the filament forming and enzymatic properties of the FliC(XynA) fusion protein. Our experiments show that replacing the D3 domain of flagellin with an enzyme protein using appropriate linkers can result in a functional fusion product that exhibits polymerization ability and catalytic activity as well. Recent studies have demonstrated the versatility of employing bioengineered flagella for the generation of a variety of nanoparticle arrays and nanotubes [23]–[26]. Our approach opens up the way for creation of building blocks for flagella-based filamentous nanostructures with designed catalytic properties.
